# Prevention of depression through nutritional strategies in high-risk persons: rationale and design of the MooDFOOD prevention trial

**DOI:** 10.1186/s12888-016-0900-z

**Published:** 2016-06-08

**Authors:** Miquel Roca, Elisabeth Kohls, Margalida Gili, Ed Watkins, Matthew Owens, Ulrich Hegerl, Gerard van Grootheest, Mariska Bot, Mieke Cabout, Ingeborg A. Brouwer, Marjolein Visser, Brenda W. Penninx, on behalf of the MooDFOOD Prevention Trial Investigators

**Affiliations:** Institut Universitari d’ Investigació en Ciències de la Salut (IUNICS/IDISPA), Rediapp, University of Balearic Islands, Carretera de Valldemosssa km 7,5, 07071 Palma de Mallorca, Spain; Department of Psychiatry and Psychotherapy, University Leipzig, Medical Faculty, Leipzig, Germany; Department of Psychology, University of Exeter, Exeter, UK; GGZ inGeest and Department of Psychiatry, EMGO+ Institute for Health and Care Research, VU University Medical Center, Amsterdam, The Netherlands; Department of Health Sciences and the EMGO+ Institute for Health and Care Research, Faculty of Earth and Life Sciences, Vrije Universiteit Amsterdam, Amsterdam, The Netherlands; Department of Internal Medicine, Nutrition and Dietetics, VU University Medical Center, Amsterdam, The Netherlands

**Keywords:** Depression, Overweight, Multi-nutrient supplements, Diet, Food behavioral activation, Food behavior, Prevention, Randomized controlled trial

## Abstract

**Background:**

Obesity and depression are two prevalent conditions that are costly to individuals and society. The bidirectional association of obesity with depression, in which unhealthy dietary patterns may play an important role, has been well established. Few experimental studies have been conducted to investigate whether supplementing specific nutrients or improving diet and food-related behaviors can prevent depression in overweight persons.

**Method/Design:**

The MooDFOOD prevention trial examines the feasibility and effectiveness of two different nutritional strategies [multi-nutrient supplementation and food-related behavioral change therapy (FBC)] to prevent depression in individuals who are overweight and have elevated depressive symptoms but who are not currently or in the last 6 months meeting criteria for an episode of major depressive disorder (MDD). The randomized controlled prevention trial has a two-by-two factorial design: participants are randomized to daily multi-nutrient supplement (omega-3 fatty acids, calcium, selenium, B-11 vitamin and D-3 vitamin) versus placebo, and/or FBC therapy sessions versus usual care. Interventions last 12 months. In total 1000 participants aged 18–75 years with body mass index between 25–40 kg/m^2^ and with a Patient Health Questionnaire-9 score ≥ 5 will be recruited at four study sites in four European countries. Baseline and follow-up assessments take place at 0, 3, 6, and 12 months. Primary endpoint is the onset of an episode of MDD, assessed according to DSM-IV based criteria using the MINI 5.0 interview. Depressive symptoms, anxiety, food and eating behavior, physical activity and health related quality of life are secondary outcomes. During the intervention, compliance, adverse events and potentially mediating variables are carefully monitored.

**Discussion:**

The trial aims to provide a better understanding of the causal role of specific nutrients, overall diet, and food-related behavior change with respect to the incidence of MDD episodes. This knowledge will be used to develop and disseminate innovative evidence-based, feasible, and effective nutritional public health strategies for the prevention of clinical depression.

**Trial registration:**

ClinicalTrials.gov. Number of identification: NCT02529423. August 2015.

**Electronic supplementary material:**

The online version of this article (doi:10.1186/s12888-016-0900-z) contains supplementary material, which is available to authorized users.

## Background

Depression and obesity are common conditions that have been consistently associated. Evidence-based research from epidemiological and clinical trials suggests a bidirectional link between being overweight and psychological health. Community-based cross-sectional studies found that depression is associated with an 18 % increased odds of being obese [17]. Longitudinal studies revealed that obesity at baseline increases the risk for onset of depression at follow-up by 55 %, and that depression at baseline increases the risk for onset of obesity at follow-up by 58 % [42].

One potential mechanism linking depression and obesity is an unhealthy diet. Consumption of processed foods such as fried foods, refined grains, and refined sugars is associated with both depression and obesity, while eating a more traditional healthy Mediterranean-style diet rich in fruits, vegetables, legumes, olive oil, fish and whole grains is associated with reduced depression [1, 11, 32, 53, 55, 56, 66–69]. These findings have also been observed in people with diabetes [18] and in older people [12]. These findings may suggest that more unhealthy food-related behavior is linked to mood states. Environmental, mood-related and food-related cues like emotions, distraction, food exposure or cooking skills can all influence the dietary intake of individuals. Empowerment to cope with these cues has been associated with positive mental health [44]. Because associations between affective states and eating behaviors have been found, frequent adherence to food-related behavior guidelines can result in a more healthy and regular dietary daily routine, higher ability to self-regulate dietary intake and increasing engagement with food as a positive activity [58].

In addition to effects of whole diet patterns, observational as well as meta-analytic evidence suggest that some specific nutrients, especially omega-3 fatty acids, folic acid, vitamin D3, selenium and calcium could have impact on depression as well [46, 49, 50, 65]. Several systematic reviews [4, 22, 41, 71] but not all [10, 54] suggest that omega-3 fatty acids may be effective at reducing depression or depressive symptomatology. A recent meta-analysis suggests that doses in the range 0.6–4.4 g of eicosapentaenoic acid (EPA) plus 0.2–2.2 g of docosahexaenoic acid (DHA) might be efficacious relative to placebo in reducing depressive symptoms in patients with depression [28]. Individuals with lower intakes of vitamin B were found to be more likely to be diagnosed with depression and to experience recurrent depressive episodes [5, 76]. Higher intakes of vitamin B6 in women and higher intakes of B12 among men were found to reduce major depressive disorder (MDD) risk [27]. Low levels of vitamin D were found in people with depression compared with controls [2, 46], in people with higher depression scores [30, 47], and were associated with higher depressive symptoms especially in persons with a previous history of depression [29]. In a male only sample, Black et al. [9] found an inverse association between serum 25-hydroxyvitamin D concentrations and symptoms of depression. Several trials have also found that vitamin D supplementation improves mood scores [40] and reduces depressive symptoms in obese people [33]. However, other controlled trials have found no benefit of supplementation on depressive symptoms in the general population [8, 19, 37]. Depression was significantly associated with low selenium blood levels [23, 26, 31] and low levels of dietary selenium were also associated with an increased risk for major depressive disorder [60]. Low dietary calcium was associated with self-rated depression in middle-aged women [6]. High intake of yogurt and calcium was associated with a lower prevalence of depressive symptoms during pregnancy in a cross-sectional study [48] and dietary intake of calcium was also associated with lower prevalence of depressive symptoms in workers [45].

The well-established epidemiological relationships between diet and depression and the positive results of several intervention studies raise the possibility that diet and nutrition may offer key modifiable targets for the prevention of MDD [56, 70]. Prevention can help to lessen the global burden of the depressive disease [16], and cost-effective interventions are required. Dietary and food related behavior interventions are potentially desirable, effective, pragmatic, scalable, and non-stigmatizing public health prevention strategies for depression. However, to date, few studies have directly manipulated diet and food-related behavior alone to examine their effects on preventing depression, leaving unresolved whether a true causal association exists from diet to depression [61] and none thus far have specifically targeted high-risk, overweight individuals.

Interventions focusing on changing food-related behaviors and shifting habitual eating patterns are one way to change diet and to have an influence on the onset of depressive episodes. Behavioral interventions can act as a selective prevention method targeting individuals or groups that are at higher risk of developing depression. An uncontrolled case series indicated the potential value of combining Behavioral Activation (BA) [43] with brief nutrition counselling among individuals with co-morbid MDD and obesity to both reduce weight and symptoms of depression [59]. BA focuses on increasing positive activities, reducing avoidance, and building routines and healthy behaviors to reduce depression. BA is an evidence-based psychological approach for depression which has demonstrated to be an effective intervention for depression in RCTs [15]. It includes detailed analysis of the antecedents and consequences of behavior in order to effectively change habitual behaviors, making it amenable to support the adoption of a healthy Mediterranean-style diet and food-related behavior. However, to date, combining BA with nutritional goals has not been tested in a controlled trial or applied to the prevention of depression.

Long-term depression prevention trials focusing on foods, nutrients and food-related behavior are currently lacking. Furthermore, studies have not fully delineated the relative influence of whole diet versus specific nutrient change on depression outcomes. Such studies are essential to determine whether it is feasible and effective to change food intake, specific nutrient intake, and/or food-related behavior for the prevention of depression. To address the gaps in the literature identified above, the Multi-country cOllaborative project on the role of Diet, Food-related behavior, and Obesity in the prevention of Depression (MooDFOOD) prevention trial will be conducted.

### Objective

The primary objective of the MooDFOOD prevention trial is to examine the feasibility and effectiveness of two different nutritional strategies to prevent a new episode of MDD in high-risk overweight persons with subsyndromal symptoms of depression. A secondary objective is to examine potential mediators and moderators of the beneficial effects (if any) of these nutritional strategies.

## Methods

### Study design

A 2 × 2 factorial randomized controlled prevention trial will be conducted to compare two nutritional strategies: a multi-nutrient supplement and a food-related behavioral change (FBC) intervention. A total of four intervention groups will be created: (1) placebo supplement group; (2) placebo supplement + FBC group; (3) multi-nutrient supplementation group; (4) multi-nutrient supplementation + FBC group.

### Recruitment and study settings

**Fig. 1:**
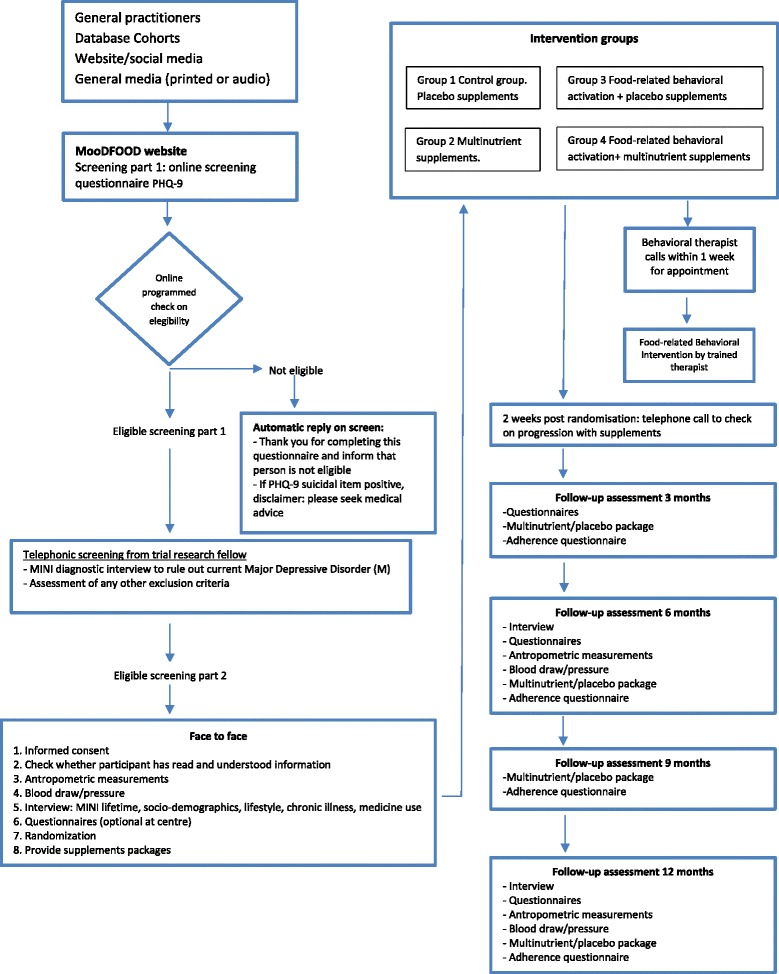
MoodFood participants flow-chart

One thousand subjects will be recruited at four study sites in four different European countries (The Netherlands: GGZ inGeest and Vrije Universiteit Amsterdam, Amsterdam; United Kingdom: University of Exeter; Germany: University of Leipzig, Spain: University of Balearic Islands, with 250 subjects recruited per site) (see Fig. 1 for recruitment process).

The recruitment strategy includes websites; local advertisements in social media; mailings to registered subjects in the general practice setting or in other registers (e.g. city registers); invitation letters, MooDFOOD brochures and posters in public areas, articles in local newspapers, press releases to the national press as well as recruitment via other studies and trials conducted at the four sites.

### Eligibility criteria

Inclusion criteria for the study are that participants will be adults aged 18 to 75 years old, meet criteria for being overweight or obese (body mass index (BMI) between 25–40 kg/m^2^) and report subsyndromal symptoms of depression as operationalized by a Patient Health Questionnaire (PHQ-9) score of at least 5. Overweight individuals with elevated symptoms of depression will be recruited because both of these variables increase vulnerability for subsequent MDD episodes. This will increase the potential base rate of incidence of depression during the follow-up period, and, thereby, the sensitivity of the prevention study. Furthermore, a nutritional intervention has greater scope to be beneficial in this group, as both obesity and depression are associated with unhealthy diet and unhealthy food-related behaviors.

Because this is a prevention trial, rather than an acute treatment trial, participants will be excluded at baseline if presenting with a current episode of (including the past 6 months) MDD (according to psychiatric DSM-IV criteria), as determined in a structured MINI International Neuropsychiatric Interview 5.0 (MINI 5.0). Other exclusion criteria include: Current (in past 6 months) use of antidepressant drugs or psychological interventions; Current eating disorder; History of psychosis, bipolar disorder, substance dependence or other severe, psychiatric disorder that requires specialized clinical attention; History of or planned bariatric surgery; Currently pregnant or breastfeeding; Current severe, life-threatening physical disease (assessed using self-report); Severe cognitive impairment sufficient to limit the conduct of the study as assessed through research staff evaluation of participant’s ability to complete the screening instruments in an adequate manner; Currently adhering to supervised behavioral interventions that interfere with MooDFOOD prevention trial interventions; Unwilling to stop using specific dietary supplements that are used or are competing with the MooDFOOD prevention trial multi-nutrient intervention.

### Screening

Participants who are interested in the study are directed to the online screening tool on our study website [http://www.moodfood-vu.eu], which provides further information and includes questionnaires to assess key eligibility criteria such as age, BMI and depressive symptoms (PHQ-9). Those individuals who pass the screening phase and indicate an interest in the study will be contacted for a more detailed telephone interview to further explain the study and confirm eligibility.

The telephone interview consists of brief screening questions on mental and physical health to check the eligibility for the trial (see Table 1). The researcher will provide the applicant with an overview of the trial and conduct the MDD section of the MINI clinical interview to assess whether individuals meet the exclusion criterion for MDD (current or in the past 6 months). Applicants who satisfy the eligibility criteria will be informed about the study design and enrolled in the prevention trial. Where applicants cannot participate because they have a psychiatric disorder, they will be advised to discuss this with their General Practitioner, and contact details of organisations for further support are offered if appropriate. Additionally, any suicidal risk reported during the interview is assessed using a well-established protocol to ensure that appropriate clinical support is provided.

**Table 1:** Inclusion and exclusion criteria of the MooDFOOD prevention trial

Inclusion criteria	
18 to 75 years old	
Body mass index between 25–40 kg/m^2^	
A PHQ-9 score of at least 5.	
Exclusion criteria	
Current (including the past 6 months) Major depressive Disorder (DSM-IV criteria, M.I.N.I. 5.0 interview).	
Current (in past 6 months) use of antidepressant drugs or psychological interventions	
Current eating disorder	
History of psychosis, bipolar disorder, substance dependence or other severe psychiatric disorder	
History of or planned bariatric surgery	
Currently pregnant or breastfeeding	
Current severe, life-threatening physical disease	
Severe cognitive impairment	
Currently adhering to supervised behavioral interventions that intervene with MooDFOOD interventions	
Unwilling to stop using specific dietary supplements competing with the MooDFOOD multi-nutrient intervention.	

*Notes*: *PHQ-9* patient health questionnaire, *MINI-5.0* MINI International Neuropsychiatric Interview 5.0

### Consent procedure, baseline and follow-up assessments

All eligible participants will then be invited to visit the research center for a face-to-face baseline assessment. The assessment takes place after written informed consent is provided, and consists of the Depression section of the MINI 5.0 to assess lifetime history of depression and a detailed interview, physical measurements (height, weight, waist circumference, blood pressure) and provision of self-administered questionnaires (see outcome measures and Table 2).

**Table 2:** Measurements and Endpoints of the MooDFOOD prevention trial

			Follow-up (in months)			
Interview		Baseline	3	6	9	12
Informed Consent		✓				
Socio-demographics	Sex, Date of Birth, civil status, housing, education, job, income	✓				
MINI (V5.0)-Present MDE	Assessment of MDE according to the definitions/criteria of DSM-IV	✓	✓	✓		✓
MINI (V5.0)-Lifetime MDE	Historical assessment of MDE according to the definitions/criteria of DSM-IV	✓				
Depression history of family	Medical records	✓				
Chronic Illness	History of Chronic illnesses	✓				
Chronic Illness follow up	Has there been a change in health?		✓	✓		✓
Medication/supplement use	Medications and supplements used in last months	✓		✓		✓
Smoking	Smoking status	✓		✓		✓
Alcohol Use Disorders Identification test (AUDIT)	Alcohol disorders	✓				✓
Compliance	Morisky Adherence Scale		✓	✓	✓	✓
Physical Measurements						
Blood pressure		✓	✓	✓		✓
Anthropometrics	Weight, height, waist circumference	✓	✓	✓		✓
Blood collection	Multi-nutrient adherence, Health indicators, markers of disease	✓		✓		✓
Self-administered questionnaires						
PHQ-9	Screening of mental health disorders	✓	✓	✓		✓
IDS-SR30	Depressive symptoms	✓	✓	✓		✓
GAD-7	Anxiety symptoms	✓	✓	✓		✓
Euro-Quol-5D-5 L	Health-realted quality of life	✓	✓	✓		✓
TEFQ-R18	Eating behavior: cognitive restraint, uncontrolled eating and emotional eating	✓	✓	✓		✓
The Stunkard figure rating scale	Body weight perception (body image and perceived body size)	✓	✓	✓		✓
SRBAI	Automaticity of good and bad health habits and Mediterranian diet	✓	✓	✓		✓
BADS	Behaviour functionality: activation avoidance/rumination, work/school impairement, and social impairement	✓	✓	✓		✓
SBQ	Sendentary behaviour	✓	✓	✓		✓
SQUASH	Physical activity	✓	✓	✓		✓
Food behaviour and Sutainability	Meal pattern, practices, cooking and shopping, mindful eating, sustainability	✓	✓	✓		✓
FFQ-GA2LEN	Food frequency questionnaire measuring food intake	✓	✓	✓		✓

*Notes*: *MINI-5.0* MINI International Neuropsychiatric Interview 5.0, *PHQ-9* patient health questionnaire, *IDS-SR30* inventory of depressive symptoms self-rated, *GAD-7* generalized anxiety disorder-7, *Euro-Quol-5D-5 L* EuroQol instrument EQ-5D-5 L, *TEFQ-R18* three-factor eating questionnaire, *SRBAI* self-report behavioral automaticity index, *BADS* behavioral activation for depression scale, *SBQ* sedentary behavior questionnaire, *SQUASH* short questionnaire to assess health-enhancing physical activity, *FFQ-GA2LEN* food frequency questionnaire

After the baseline measurement, the participant will be randomised to one of the four intervention groups. Participants are given their first 3-month supply of pills (multi-nutrient supplement or placebo) with oral and written instructions on how to use them. Participants randomized to FBC will receive a telephone call from the behavioral therapist within one week for an appointment. Two weeks after randomization all participants will receive a telephone call to monitor the progression with supplements and to improve compliance.

All interventions will last 12 months. Follow-up assessment will be conducted by researchers unaware of the randomization status at 3, 6, and 12 months. Table 2 gives an overview of all measurements. Adherence to the supplement use will be measured at 3, 6, 9, and 12 months. In a subsample of trial participants (at least *n* = 50 per intervention arm) blood samples will be collected at baseline, at 6 and 12 months assessments for the investigation of nutrient concentrations to monitor multi-nutrient supplement compliance. Three-month supplies of pills will be provided at the 3, 6 and 9 month contacts.

### Randomization, allocation and masking

Participants will be randomized with equal probability (in a 1:1:1:1 ratio) to the four intervention arms. Permuted block randomisation will be employed using off-site blind computer generated quasi-random numbers obtained prior to trial commencement (https://www.sealedenvelope.com). Randomisation will be stratified according to recruitment site (four sites, enabling a steady flow to intervention groups) and participants’ history of depression status at the baseline assessment (lifetime history of MDD versus no MDD), determined by use of the MINI 5.0 interview for depression. The online randomization procedure will generate a unique code that is linked to both the multi-nutrient supplement (active nutrients or placebo) and FBC intervention status (yes/no).

Researchers will dispense the multi-nutrient supplements to the participant using the unique code. The placebo and supplement pills are identically packaged and indistinguishable from one another except through the unique codes. Therefore participants, therapists, and researchers will be blind (triple-blind) to supplement allocation. Participants of the FBC intervention will be contacted directly by the MooDFOOD therapist, thus ensuring that researchers, assessing the primary outcome, additionally remain blind (single-blind) to behavioral intervention status. All assessments will be conducted equally in all treatment conditions by researchers blind to randomization. To successfully maintain the blinding throughout the trial, the researchers conducting the patient assessments will be instructed to remind participants of the confidential nature of their treatment.

### Interventions

#### Multi-nutrient supplement

Participants will be asked to take the multi-nutrient supplements or placebo pills every day during one year. The placebo and multi-nutrient supplements will be plainly packaged and identical to one another in every way, with the exception of different codes on the packaging to enable blind allocation and unblinding if necessary.

The supplements will contain nutrients for which deficiency or low levels have been linked to depression risk in prior epidemiological or clinical intervention studies (see introduction). One daily supplement consists of omega 3 fatty acids in the form of 1412 mg of EPA and DHA (ratio 3:1) in a pharmaceutical form of a soft gelatine capsule of non-porcine origin with an enteric coat to prevent backflow. The other daily supplement is a multi-nutrient pill containing 100 mg of Calcium, 30 μg of Selenium, 400 μg of Folic acid (folate) and 20 μg of vitamin D3.

To match the active nutrient supplements, there will also be two placebo pills: a sunflower oil capsule with similar filling material and colour as the fatty acid capsule and a second pill with similar colour and shape as the multi-nutrient supplement containing Microcrystalline cellulose; Corn Starch; Polyvinylpyrrolidone; Crosslinked carboxymethylcellulose Sodium; Magnesium Stearate and Magnesium Silicate.

#### Food-related behavioral change (FBC)

The FBC intervention will consist of up to 21 sessions (up to 15 individual sessions of 30 min, provided in single or double (1 h) meetings; in addition to up to 6 group-based sessions lasting approximately 1 h). Individual sessions with the therapist will occur weekly at first and then every two weeks, and the final 6 sessions in groups of up to 10 people will take place monthly and then bimonthly. Different sessions may be delivered over the telephone or via web chat software such as Skype™. The treatment is manualized, with a handbook outlining the general principles of the intervention and mapping out the required content of each session. The intervention includes detailed analysis of each participant’s behavior to determine idiosyncratic triggers and functions of unhelpful (e.g., mood-related snacking) and helpful food-related behavior, in order to reinforce helpful behaviors and to implement effective alternatives to unhelpful behaviors, building on behavioral approaches proven effective in depression [15, 43]. The FBC intervention incorporates the standard approaches of BA including self-monitoring, functional analysis, and activity scheduling, with a focus on reducing avoidance and changing habits, with specific nutritional advice related to mood focused on building up a healthy Mediterranean style diet and improving food-related behaviors (e.g. regular meals per day) [39, 57]. At each site, clinicians familiar with behavioral activation principles (e.g., clinical psychologists, psychological wellbeing practitioners) will receive specific training in the FBC and on diet and food-related behavior by a nutritional scientist (IB) and will deliver the intervention. A dietician will be available for consultation across all sites.

#### Intervention fidelity

To ensure good adherence and competence to the FBC intervention, all sites will conduct regular supervision of therapists, in which cases will be discussed and audio/video reviewed. 5 % of all recorded sessions across all sites will be rated by blind independent raters against relevant fidelity and competence criteria, selected at random throughout the course of therapy (with an emphasis on selecting earlier rather than later sessions). All therapists will complete an initial intensive 4-day training in FBC, led by an experienced BA therapist (ERW) and will have to demonstrate competence and fidelity in an observer-rated role-play to receive certification to deliver therapy for the trial. A therapy template form will also guide therapists in session delivery, recording what participants received and guide supervision. A follow-up 2-days training session involving all therapists will be performed. Adherence to the manuals will be rated by using a checklist to assess the presence of key therapy components in general and for each specific session. Competence will be rated by using an adjusted Cognitive Therapy Rating Scale for the FBC intervention. Kappa statistics on inter-rater reliability will be reported.

### Outcomes

#### Primary outcome

The primary outcome of this prevention trial will be the 12-month onset of an episode of MDD, defined according to the standard psychiatric Diagnostic and Statistical Manual of Mental Disorders, Fourth Edition (DSM-IV) criteria using the Depression section of the MINI 5.0 at 3, 6, and 12 months. The MINI 5.0 is a brief standardized diagnostic interview used to assess disorders described in the DSM-IV [73].

#### Secondary outcomes

Secondary outcomes will be assessed at baseline, 3, 6, and 12 months after start of study by using the following measures: PHQ-9 [38] and the self-rating version of the Inventory of Depressive Symptomatology (IDS30-SR) [64] will be used to assess depressive symptoms. The Generalized Anxiety Disorder-7 (GAD-7) questionnaire will be used to assess anxiety symptoms [74]. Health-related quality of life will be assessed by the EuroQol instrument EQ-5D-5 L (EuroQol Group, 1990). Food-related behavior will be assessed by the Three-factor Eating Questionnaire (TFEQ-R18) [36], and a MooDFOOD project developed Food Behavior Questionnaire that assesses meal pattern, snacking behavior, and other food-related behaviors in the past two weeks, such as mindful eating, cooking and shopping skills. Food intake will be assessed by the GA2LEN-FFQ questionnaire [24]. Physical activity and sedentary behaviour will be assessed by the Short Questionnaire to Assess Health-Enhancing Physical Activity (SQUASH) [77] and the Sedentary Behavior Questionnaire (SBQ) [63]. Body weight perception will be assessed by the Stunkard figure rating scale [75].

Mediating/moderating variables: Some secondary outcomes (food intake and food-related behavior, body image perception, and psychological eating behavior) will also be considered as mediating variables to explain impact on the primary outcome. Additional mediating mechanisms will also be assessed: Adherence to pills will be assessed with an adapted version of the Morisky Adherence Scale, a structured four-item self-reported adherence measure [51]; Anthropometry (measured height, body weight, waist circumference according to standardized measurement protocols and calculated BMI); Body composition (fat mass, fat-free mass and percentage body fat) will be accurately assessed using whole body air-displacement plethysmography (BodPod) in participants from one site (Exeter); and blood pressure. Patterns of behavior will be assessed by the Behavioral Activation for Depression Scale (BADS) [35] a 25-item questionnaire measuring changes in avoidance and activation over the course of behavioral activation, and the Self-Report Behavioral Automaticity Index (SRBAI) [25] adapted to measure changes in patient-identified healthy and unhealthy mood and food-related habits. Somatic health status will also be assessed in terms of self-report of somatic/chronic diseases, smoking, alcohol consumption and medication/supplements use at 6 and 12 months.

In a subsample of trial participants, blood samples will be collected at baseline, and after 6 and 12 months to measure blood levels of the nutrients provided with the multi-nutrient supplements, as a manipulation check for adherence to the multinutrient supplements/diet change.

The following descriptive variables and potential moderating variables will be assessed only at baseline: country, age, gender, educational level, family’s medical history, household income, earlier psychiatric history and somatic health status participants through self-report of somatic diseases.

### Sample size

Effective preventive interventions in high-risk groups are found to reduce the onset of depression by as much as 25–50 % [7]. We have therefore powered this trial at the conservative end of this range, by assuming a difference of 33 % in the 12-month cumulative incidence rate of DSM-IV episodes of MDD between the presence of an active condition (estimated 12-month cumulative incidence of MDD 20 %) versus its absence (estimated 12-month cumulative incidence of MDD 30 %) (i.e., FBC versus no FBC; multinutrient supplement versus placebo). The 2×2 factorial design is efficient for sample size and power and enables us to test for potential synergistic interactions between interventions [13, 14]. Assuming a difference between an active condition (20 % incidence of MDD) and a control condition (30 % incidence of MDD), 392 participants would be needed to evaluate the main effect of each nutritional intervention assuming a 2-sided test at α = .05 and a power of (1 − β) = 0.90. This means that 196 participants in each of the four cells would be needed. Assuming a follow-up attrition rate of 22 %, a total of 250 participants per cell would be sufficient. A reduction in sample size to 147 participants in each cell would reduce the power to detect an effect of this magnitude to 80 % power.

### Statistical analysis plan

Statistical reporting will follow the CONSORT standards [72]. Missing data will be inspected and handled via full information maximum likelihood (FIML) or multiple imputations (MI) as appropriate. The primary analyses will be Intention-To-Treat (ITT) analyses [78]. Subsequent analyses will use the Complier Average Causal Effect (CACE) analysis [3, 20] to provide an estimate of a treatment effect taking into account adherence and compliance with the treatment, whilst retaining the benefits of randomization.

Following the 2×2 factorial design of the trial, we will test for the effects of the two nutritional strategies separately as well as combined using logistic regression models for the dichotomous primary outcome (12-month cumulative incidence of episodes of major depressive disorder). The secondary outcomes will be examined using mixed models or generalized estimating equations (GEE) to assess the longitudinal change in depressive symptoms, and other secondary outcomes. We will adjust the analyses for site and history of MDD to account for the two stratification variables used in the randomization [34]. The results from the trial will be prepared as comparative summary statistics with 95 % confidence intervals. *P*-values <0.05 will be considered statistically significant.

For the secondary objectives, we will conduct mediation analyses to gain insight into mechanisms that could explain the potential effect of the nutritional interventions on depression. We will use modern causal inference methods using parametric regression models to assess mediation effects [21]. This involves using parametric regression models to test for mediation of the dietary interventions on depression through changes in the mediating variables. In addition, we will investigate potential moderation of the nutritional strategies on depression by site, age, sex, educational level, comorbid illness, initial BMI, and depression history.

### Organization, and quality assurance and data management

Compliance with the multi-nutrient supplement intake will be assessed using questionnaires. In addition, participants will be instructed to return the supplement containers once every three months. These jars will be weighed to determine the number of unused supplements. Any positive or adverse effects of the supplements will be evaluated at each assessment. Attendance of the FBC sessions will be recorded by the research staff.

Research data will be collected in an anonymised manner by means of computer-assisted interviews and online questionnaires. Questionnaires from respondents who prefer a paper version will be entered into the system by a research assistant. Each site uses an administrative database to ensure timely assessments. The central data-management team uses de-identified backups for the monitoring of the overall progress and data quality. Ultimately, a comprehensive dataset will be produced that includes data from all four research sites.

A Trial Steering Committee (TSC) was created with the following activities: To monitor and supervise the progress of the trial towards its objectives; To review at regular intervals relevant information from other sources (e.g., other related trials and research groups); To consider recommendations of the Research Ethics Committee; To determine if interim analyses of trial data should be undertaken; To consider the data from interim analyses; To consider any safety issues for the trial and recommend appropriate contingencies; To consider any requests for release of interim trial data; In the event of further funding being required, to provide to the Funder appropriate information and advice on the data gathered to date without jeopardising the study.

The members of the TSC will be relevant experts in the areas of knowledge of the project: Marijke Bremmer (Netherlands), ethical advisor; Adriaan Hoogendoorn (Netherlands), statistician; Simon Gilbody (United Kingdom), psychiatrist and clinical and research expertise and Steffi Baumann (Germany), patient/lay representative. M Bremmer will be the chair of the committee. A person from Exeter group will assist and help with the monitoring of the TSC. All TSC members will sign a Non-Disclosure- Agreement.

### Trial status

The Trial was registered in ClinicalTrials.gov. Title of registration: Multi-country cOllaborative Project on the rOle of Diet, FOod-related Behaviour, and Obesity in the Prevention of Depression (MoodFOOD). Number of identification: NCT02529423. (www.clinicaltrials.org). August 2015. Recruitment commenced in August 2015 and is ongoing.

## Discussion

Strategies to prevent the onset of depression are a public health priority [52, 62]. This MooDFOOD Prevention Trial provides the first direct test of the use of two distinct nutritional strategies (multi-nutrient supplements and food-related behavioral change) to prevent the incidence of depression in a large-scale sample of overweight individuals with subsyndromal levels of depression. As such, this trial is relevant to tackling two major global health challenges: depression and obesity. Epidemiological and clinical intervention data indicate that food intake and food-related behavior may be involved in risk for depression, especially in people who are overweight, but to date the majority of data is observational and making firm conclusions on causality has been difficult to date. Large-scale intervention studies to establish the true impact of food intake and food-related behavior on the development of depression are therefore needed.

The proposed trial will examine the impact of nutritional strategies across a large sample of 1000 participants, across four distinct European states, potentially enabling generalization to the wider European population. This trial seeks to obtain scientific knowledge on the link between diet and depression at a much higher level of evidence (level A) than most previous studies. This knowledge on causal relationships will enable the development of evidence-based nutritional strategies for the prevention of depression. Furthermore, the repeated measurement of potential intermediate variables over 12 months, will contribute to testing the causal mechanisms underlying the link between diet and depression.

The use of nutritional strategies may provide an effective, acceptable, and highly scalable approach for the prevention of depression in overweight adults. Widespread implementation is a key factor in prevention, and a nutritional approach may provide a valuable tool in improving access to evidence-based interventions. It is hoped that this large-scale trial will enable the development of evidence-based and innovative nutritional strategies for the prevention of depression, thereby importantly contributing to better quality of life and healthy aging for EU citizens. Please, see Additional file 1 to know the dissemination policy adopted for the consorcium with specific guidelines on disseminations of results and nutritional strategies to general and specialized public. 

## Abbreviations

BA, behavioral activation; BADS, behavioral activation for depression scale; BMI, body mass index; BodPod, body air-displacement plethysmography; CACE, complier average causal effect; DHA, docosahexaenoic acid; DSM-IV, diagnostic and statistical manual of mental disorders, fourth edition; EPA, eicosapentaenoic acid; FBC, food-related behavioral change; GAD-7, generalized anxiety disorder-7; GEE, generalized estimating equations; IDS30-SR, inventory of depressive symptomatology; ITT, intention-to-treat; MDD, major depressive disorder; MI, multiple imputations; MINI 5.0, MINI International Neuropsychiatric Interview 5.0; MooDFOOD, Multi-country cOllaborative project on the role of Diet, Food-related behavior, and Obesity in the prevention of Depression; NIHR, National Institute for Health Research; NRES, NHS National Research Ethics Service; PHQ-9, patient health questionnaire; RCT, research control trial; SBQ, sedentary behavior questionnaire; SQUASH, short questionnaire to assess health-enhancing physical activity; SRBAI, self-report behavioral automaticity index; SRBAI, self-report behavioral automaticity index; TFEQ-R18, three-factor eating questionnaire; TSC, Trial Steering Committee

## Additional files

Additional file 1: Dissemination policy. Dissemination plan of results and developed nutritional strategies. (DOCX 17 kb) Additional file 2: The MooDFOOD prevention trial investigators. A list of all researchers and clinicians participating in the randomized controlled prevention trial by center and in the project office. (DOCX 15 kb) Additional file 3: Consent of participation. Consent to participate form. (PDF 240 kb) Additional file 4: Standard Protocol items: Recommendations for Interventional Trials. SPIRIT 2013 guidelines addressing information related to the randomized controlled prevention trial. (DOC 121 kb)
