# Supplementary material for: Prevention of depression through nutritional strategies in high-risk persons: rationale and design of the MooDFOOD prevention trial
Source: BMC Psychiatry. 2016 Jun 8;16:192. doi: 10.1186/s12888-016-0900-z (PMC4898322; doi:10.1186/s12888-016-0900-z)
Supplement: Additional file 1: — Dissemination policy. Dissemination plan of results and developed nutritional strategies. (DOCX 17 kb) [file 12888_2016_900_MOESM1_ESM.docx]

**Appendix 1. Dissemination Policy**

**Objectives**

Objective 1.
A collaborative working platform will be created for use in all work packages and produce and disseminate information on MooDFOOD, its objectives, research approaches and results to the project’s target audiences: the scientific community, media, general public, policy makers, health professionals and relevant scientific societies, relevant industries (including SMEs), consumer organizations. Focus will be on both European and International dissemination.

Objective 2.
To develop a strategy for the translation of the scientific outputs and results into practical tools (guidelines, policy documents, research methods etc.) to prevent depression through nutritional strategies.

Objective 3.
To promote the use of project results and implementation tools by European and International stakeholders and target groups.

**Description of work and role of partners**

Facilitate optimal communication within and outside the consortium and build a road map for dissemination of the developed nutritional strategies including a strong communication and marketing strategy. Translate scientific results and data into dissemination materials, with actions being coordinated by a communication/dissemination plan. Dissemination materials (online and print) will be translated where possible via EASO’s network in 30 European countries and partner networks.

1. To create a collaborative working platform for use in all work packages and develop a dissemination and communications plan.
   Outline key strategies for project dissemination and communications, for both internal (project partners) and external stakeholders. Define key messages to disseminate, identify appropriate dissemination tools and channels, define the target audiences (e.g. the European and international scientific communities, policy makers, relevant scientific societies, EU citizens, Industry), and incorporate a strategy for media monitoring, (classical and social media). Update the plan after the 2nd General Assembly meeting and then annually. Produce a dissemination report at the end of the project.
2. Development and maintenance of project identity and project website
   a) Create a corporate identity in order to ensure a common graphic line for all communications material produced by the consortium. b) A specific website for the project will be established and maintained for the duration of the project and until 3 years after its completion. Its structure will be: 1) An external website (accessible to everyone) that will be the main information resource of the project (describing project objectives, structure and results), coordinated by EASO. 2) An additional internal platform (restricted area), designed and used to share preliminary results and internal documents (e.g. working papers, deliverables progress, calendar of events) amongst project partners and the European Commission Officers, and which will also contain the data management application (see WP4).
3. Scientific exchange and dissemination.

a) Scientific papers from the various WPs will be published in relevant journals throughout the duration of the project. A scientific summary paper will be published at the end of the project for which open access will be purchased. b) Relevant outcomes, findings, key recommendations, implementation tools and the final report will be disseminated to the scientific community in a series of society e-newsletters (EASO, EAAD and GAMIAN plus relevant project partners) and e-newsletters on the project website and partner networks. c) The project will connect with relevant European and International networks via project workshops. Two interim workshops will take place as satellite symposia at relevant congresses. A final project workshop (the aim of which is to present final results to key target audiences) will be organised as a stand-alone meeting in Brussels. d) All project partners will develop and expand existing networks to allow wide scientific dissemination. A database of interested parties will be created as a dissemination channel. The links that EASO (and project partners) have with the EU Platform for Action on Diet, Physical Activity and Health will be built on to disseminate results.

4. Translate nutritional strategies to remedial actions.
Translation of nutritional strategies into practical tools for stakeholders which include but are not limited to: a) Treatment guidelines and practical tools for health professionals to prevent depressive symptoms in depressed and non-depressed consumers; b) Evidence-based nutritional guidelines for EU citizens including the whole diet as well as nutrient supplement use; c) Food product development recommendations and scientific evidence supporting health claims for the food industry; d) Policy advice and recommendations to promote healthy eating and prevent depression for individual Member States and the EU.

5. Classical Dissemination of remedial actions.
a) Two Project leaflets (in English) will be produced, one presenting the main elements of the project (at the beginning) and the other summarising project achievements (at the end), to be distributed through partners networks and at relevant conferences/events. b) Press releases on the project and its results will be written in English and translated into relevant languages, and sent to European and international press outlets and to national journalists. c) Print and online dissemination of the translated nutritional strategies. d) A final conference at the end of the project will be organised for key target audiences. A summary of the project results will be made available for stakeholders before the end of the project.

6.Non-classical dissemination (social media) of remedial actions.
To increase outreach and types of audience, various online tools will be used to promote further the project results in an accessible, direct and understandable, yet scientifically sound fashion. a) Two podcast series based on interviews/presentations with key partners during project workshops and one webinar of the final conference. These will be disseminated via the project website and at relevant scientific meetings (for example EASO’s ECO, IUNS and EPA and mainstream internet outlets such as YouTube). c) A MooDFOOD Twitter account and/or Facebook page to ensure social media presence, reach of different target audiences and wider dissemination. d) Mutual links will be established with project partners’ websites and organisations working or with interest in this area. e) A MooDFOOD section will be created on www.easo.org and the websites of the dissemination partners to drive traffic to the project website.
