# Supplementary material for: Prevention of depression through nutritional strategies in high-risk persons: rationale and design of the MooDFOOD prevention trial
Source: BMC Psychiatry. 2016 Jun 8;16:192. doi: 10.1186/s12888-016-0900-z (PMC4898322; doi:10.1186/s12888-016-0900-z)
Supplement: Additional file 2: — The MooDFOOD prevention trial investigators. A list of all researchers and clinicians participating in the randomized controlled prevention trial by center and in the project office. (DOCX 15 kb) [file 12888_2016_900_MOESM2_ESM.docx]

# Appendix

**The MooDFOOD prevention trial investigators**

**MooDFOOD project coordination, VU University Amsterdam, Department of Health Sciences, the Netherlands**

Prof. Marjolein Visser, PhD – Principle Investigator of the MooDFOOD project and the MooDFOOD prevention trial

Prof. Ingeborg A Brouwer, PhD – Co-Principle Investigator of the MooDFOOD project and the MooDFOOD prevention trial

Mieke Cabout – Assistant project manager of the MooDFOOD project

**Trial Centers**

**VU Medical Center, Amsterdam, the Netherlands**

Prof. Brenda Penninx, PhD – Field Center Principal Investigator

Dr. Mariska Bot, PhD – Field Center Co-Investigator

Nadine Paans – Field Center therapist and research assistant

Carisha Thesing – Field Center therapist

Deborah Gibson-Smith – Field Center research assistant

Melany Horsfall – Field Center coordinator

Lena Weiss – Field Center research assistant

**University of Exeter, Exeter, United Kingdom**

Prof. Ed Watkins, PhD – Field Center Principal Investigator, lead for developing the MooDFOOD Food-related Behavioural Change Intervention

Dr. Matthew Owens, PhD – Field Center Co-Investigator

Dr. Amy Romijn, PhD – Field Postdoctoral Research Associate

Owain Winfield – Field Center Researcher and Therapist

Caterina Versari Molinares – Research Intern

Atikah Sapar – Research Intern

**University of Balearic Islands, Palma de Mallorca, Spain**

Prof. Miquel Roca, PhD, MD – Field Center Principal Investigator

Prof. Margarita Gili, PhD – Field Center Co-Investigator, co-lead for developing the MooDFOOD Food-related Behavioural Change Intervention

Prof. Miquel Tortella, PhD – Field Center-Co-Investigator, co-lead for developing the MooDFOOD Food-related Behavioural Change Intervention

Clara Homar Covas – Field Center Researcher and therapist

Margalida Vives Forteza – Field Center Research assistant

Adoración Castro Gracia – Field Center Research assistant

José Luis Reig – Field Center therapist

**University Leipzig, Leipzig, Germany**

Prof. Ulrich Hegerl, MD – Field Center Principal Investigator

Dr. Elisabeth Kohls, PhD – Field Center Co-Investigator

Jana Hoesel – Field Center study nurse

Ezgi Dogan, MD – Field Center research fellow

Sabrina Baldofski – Field Center therapist

Nicole Mauche – Field Center therapist

**Data Management, GGZingeest, Amsterdam the Netherlands**

Prof. Brenda Penninx, PhD – Principal investigator

Gerard van Grootheest – Data management coordinator

Bep Verkerk – Data manager

**Funding**

Funding for the MooDFOOD prevention trial was provided by the European Union FP7 MooDFOOD Project ‘Multi-country cOllaborative project on the rOle of Diet, FOod-related behaviour, and Obesity in the prevention of Depression’ (grant agreement no. 613598).
