# Supplementary material for: Prevention of depression through nutritional strategies in high-risk persons: rationale and design of the MooDFOOD prevention trial
Source: BMC Psychiatry. 2016 Jun 8;16:192. doi: 10.1186/s12888-016-0900-z (PMC4898322; doi:10.1186/s12888-016-0900-z)
Supplement: Additional file 3: — Consent of participation. Consent to participate form. (PDF 240 kb) [file 12888_2016_900_MOESM3_ESM.pdf]

MooDFOOD
PREVENTING DEPRESSION THROUGH FOOD

ID:
Blood ID:

MooDFOOD Trial CONSENT TO PARTICIPATE FORM

	Please initial
1. I confirm that I have read and understand the information sheet [version 2.2] and have had the opportunity to ask questions which have been answered to my satisfaction.	
2. I understand that my participation is voluntary and that I am free to withdraw at any time without giving any reason, without my medical care or legal rights being affected.	
3. I agree to take part in the above study, and to my GP being informed of my participation in this study and updated with information from this study relevant to my medical care.	
4. I understand that relevant sections of my medical notes and data collected during the study may be looked at by individuals from regulatory authorities or from the NHS Trust where it is relevant to my taking part in this research. I give my permission for these individuals to access my records.	
5. I understand that if I choose to withdraw from the study, data and samples already collected may be used in conjunction with this study.	
6. I agree to my data from this study being shared with other researchers after my personal identifying information has been removed.	
7. I consent to be randomly allocated (by chance, like tossing a coin) to one of the intervention groups. I am aware that I have a 75% chance of receiving at least one of the active interventions (multivitamin and mineral supplements or the lifestyle and behavioural coaching).	
**OPTIONAL**	
8. I agree to my interviews and any therapy sessions I receive being audio-recorded/video-recorded for research purposes.	
9. I agree to my contact details being added to the Mood Disorder Centre database so that I might be invited in the future to take part in depression research.	
10. I understand and agree to gift blood samples to measure nutrient levels, and other biological factors associated with obesity, vascular disease and depression for analysis now (cholesterol) and in the future for research related to this study.	
11. I consent to my anonymised samples being securely transported from the approved research bank in Exeter to the MooDFOOD laboratory in Spain for analysis for nutrient levels and cholesterol.	
12. I agree to gift blood samples for genetic (DNA and RNA) analysis which will be stored for future research and may be transferred to the ethically approved Peninsula Research Bank in the future. I understand that any samples or information on my DNA or RNA that leaves the Clinical Research Facility in Exeter will be anonymised.	
13. I agree to do the Bodpod procedure (body composition analysis).	
14. I would like to be sent information about the results of the research when they are available.	

Name of participant *(BLOCK CAPITALS)* **Date** **Signature**

- -

TO BE COMPLETED BY THE RESEARCHER

“I have explained the study to the above participant and they have indicated their willingness to take part in the study”

Name of researcher *(BLOCK CAPITALS)* **Date** **Signature**
